# The Influence of Decent Work on Sustainability Behavior in Nurses and the Mediating Role of Professionalism

**DOI:** 10.1097/jnr.0000000000000744

**Published:** 2026-05-08

**Authors:** Mohamed Ali ZOROMBA, Heba Emad EL-GAZAR

**Affiliations:** 1 College of Nursing, Prince Sattam bin Abdulaziz University, Al-Kharj, Saudi Arabia; 2 College of Nursing, Imam Mohammad Ibn Saud Islamic University (IMSIU), Riyadh, Saudi Arabia

**Keywords:** decent work, professionalism, sustainable behavior, nurses

## Abstract

**Background:**

With the increasing emphasis on sustainability in health care, it is essential to clarify how work conditions and professional identity influence sustainable behavior in nurses. Decent work environments may contribute to professionalism, which is known to foster sustainable practices.

**Purpose:**

This study was designed to investigate the relationship between decent work practices and self-reported sustainable behavior in nurses, with a focus on the mediating role of professionalism.

**Methods:**

This cross-sectional study was conducted on a sample of 206 clinical nurses. Data were collected using validated Arabic versions of the Decent Work Scale, Nurses’ Professionalism Inventory, and Self-Reported Sustainable Behavior Scale. Statistical analyses included Pearson correlation, *t*-tests, analysis of variance (ANOVA), and mediation analysis.

**Results:**

The results revealed significantly positive associations between all of the study variables. Decent work practices were shown to correlate strongly with both professionalism (*r*=.870) and sustainable behavior (*r*=.897). Also, professionalism was found to correlate positively with sustainable behavior (*r*=.823). The results of the mediation analysis confirmed professionalism to significantly mediate the relationship between decent work and sustainable behavior (indirect effect β=0.332, 95% CI [0.194, 0.447]).

**Conclusions/Implications for Practice:**

Decent work conditions enhance professionalism and, as a result, promote sustainable behavior in nurses. These findings underscore the importance of fostering supportive work environments and professionalism to achieve sustainable health care practices. Interventions that enhance decent work and professionalism may play a critical role in advancing sustainability in health care.

## Introduction

In recent years, sustainability has emerged as a crucial objective across various sectors, including health care, where the focus has increasingly shifted toward promoting environmentally, economically, and socially responsible practices. The health care sector, with its extensive use of resources and potential for significant environmental impact, has a vital role to play in the global sustainability agenda (Or & Seppänen, 2024), with related responsibilities extending to all health care professionals—especially nurses, who represent the largest segment of the health care workforce and are integral to the implementation of sustainable practices within their daily operations (O’Hara et al., 2022).

Nurses, as frontline health care providers, are uniquely positioned to influence sustainability within health care settings. Their daily activities, decisions, and interactions with patients and other health care professionals can have a profound impact on environmental, economic, and social sustainability (Luque-Alcaraz et al., 2024). For example, nurses contribute to environmental sustainability through their waste management practices, energy use, and choice of materials and products used in patient care (Schenk et al., 2023). Furthermore, advocacy by nurses for equitable and accessible health care aligns with the social dimension of sustainability, ensuring all patients receive quality care irrespective of their socioeconomic status (Hassmiller & Wakefield, 2022).

The global emphasis on sustainability in health care is reflected in initiatives such as the United Nations’ Sustainable Development Goals (SDGs), particularly SDG 3, the emphasis of which is on safeguarding healthy survival and endorsing well-being for all ages (United Nations, 2015). Nurses are central to achieving these goals through their direct patient care roles and involvement in broader health care system processes (Wakefield et al., 2021). This is where the concept of “decent work” becomes critical.

### Literature Review and Hypothesis Development

#### Decent work and professionalism in nursing

The concept of decent work, as outlined by the International Labour Organization, has gained significant attention in various sectors, including health care. Decent work is characterized by chances for creative work that deliver fair income, workplace security, and social guardrails for workers and their families (International Labour Organization, 2019). In the health care sector, particularly in nursing, decent work is essential not Z only for the well-being of nurses but also for ensuring high standards of patient care (El-Gazar, Zoromba et al., 2022).

In the context of health care workers, research findings have shown decent work conditions to be closely linked to increased job satisfaction and professional engagement. For instance, Kaan Namal et al. (2024) found nurses who perceive their work environment as supportive and aligned with the principles of decent work to be more likely to exhibit high levels of professionalism. This is because decent work environments provide the necessary resources, support, and opportunities for professional growth, all of which are critical to fostering a strong professional identity among nurses (El-Gazar & Zoromba, 2024: Xue et al., 2024).

Professionalism in nursing, encompassing the commitment to ethical practice, continuous self-improvement, and advancement of the nursing profession, is a key determinant of the quality of care provided by nurses and is closely linked to their ability to engage in reflective practice and uphold related professional standards (He et al., 2024). The relationship between decent work and professionalism may be understood through the lens of job resources theory, which posits that the availability of resources in the workplace (such as decent work conditions) enhances the motivation, engagement, and professional behavior of employees (Bakker et al., 2023). Based on the above, it is hypothesized in this research that (H1): Decent work practices relate positively to professionalism in nurses.

#### Professionalism and sustainable behavior in nursing

Sustainable behavior in nursing involves actions that contribute to the long-term well-being of patients, communities, and the environment (Shaban et al., 2024). Nurses who demonstrate high levels of professionalism are more likely to engage in sustainable practices as part of their commitment to providing high-quality care (El-Gazar & Zoromba, 2021). A mixed-method analysis by Luque-Alcaraz et al. (2024) found that nurses who prioritize professionalism in their practice are more likely to adopt sustainable behaviors.

Moreover, professionalism is associated with a continuous commitment to learning and improvement, which drives nurses to stay informed about best practices in sustainability and to integrate these practices into their daily work. This commitment to professional development aligns with the principles of sustainability, as it encourages nurses to consider the long-term impact of their actions on patients, communities, and the environment (Mlambo et al., 2021). Given the abovementioned strong theoretical and empirical linkages, it is hypothesized in this research that (H2): Professionalism in nurses relates positively to their self-reported sustainable behavior.

#### Direct impact of decent work on sustainable behavior

Decent work environments provide the physical, social, and psychological resources that enable nurses to engage in practices aligned with the principles of sustainability. In health care settings, decent work conditions such as safe working environments, adequate compensation, and work-life balance are essential to promoting sustainable behavior among nurses. Furthermore, decent work practices that promote equity and social justice can encourage nurses to advocate for practices that promote health equity and social sustainability in their communities (El-Gazar, Abousoliman, et al., 2024; Sönmez et al., 2023).

The findings reported in Xue et al. (2024) indicate that experiencing decent work conditions makes nurses more likely to be motivated to engage in work behaviors. This includes adopting sustainable practices that contribute to the long-term viability of health care resources and the well-being of patients and communities. In light of this evidence, it is hypothesized in this research that (H3): Decent work practices relate positively and directly to self-reported sustainable behavior.

While decent work may impact sustainable behavior directly, the role of professionalism as a mediating factor cannot be overlooked. The relationship between decent work and sustainable behavior is likely to be mediated by the level of nurse professionalism. This is because decent work environments foster professionalism, which in turn may drive sustainable behavior (El-Gazar, Zoromba et al., 2022; Xue et al., 2024). Professionalism may be seen as a mechanism through which the effects of decent work on sustainable behavior are realized. When nurses perceive their work environment as supportive and fair, they are more likely to develop a strong professional identity, which includes commitments to an ethical practice and sustainability (Wakefield et al., 2021). Subsequently, this professional identity motivates nurses to engage in behaviors that contribute to sustainability both in their immediate work environment and in the context of the broader health care system (Algabar et al., 2023).

The mediating role of professionalism in the relationship between decent work and sustainable behavior is supported by job resources theory, which posits that the availability of resources in the workplace enhances employee motivation and engagement, leading to positive work outcomes (Bakker et al., 2023). In this case, decent work conditions serve as a resource that enhances nurse professionalism, which, in turn, promotes sustainable behaviors. Furthermore, according to the theory of planned behavior, people’s intentions and actions are influenced by their attitudes, subjective norms, and perceived behavioral control (Ajzen, 1991). In the context of nursing, professionalism may be considered a positive attitude that influences intentions to engage in sustainable behavior. Decent work conditions provide the support and resources necessary to shape these positive attitudes, thus mediating the relationship between decent work and sustainable behavior. Based on the theoretical and empirical evidence, it is hypothesized in this research that (H4): The relationship between decent work practices and self-reported sustainable behavior in nurses is mediated by the level of nurse professionalism.

### Research Gap

Whereas these constructs have been examined individually in previous research, they are assimilated in this study into a comprehensive model using an empirical approach that highlights the interdependencies among work conditions, professional identity, and sustainability in the context of health care. By identifying the mechanisms by which decent work influences sustainable behavior, the findings are expected to offer valuable insights to health care managers and policymakers that they may apply to enhance sustainable practices in health care. Moreover, given the push toward sustainability worldwide, identifying those factors that enable nurses to contribute effectively to this goal is critical.

### Study Aim and Objectives

The aim of this study was to explore the relationship between decent work and self-reported sustainable behavior in nurses, with a focus on understanding how professionalism mediates this relationship. The research objectives were to:

1. Examine the relationship between decent work practices and professionalism in nurses.
2. Investigate the direct impact of decent work practices on sustainable behavior.
3. Assess the role of professionalism as a mediator in the relationship between decent work and sustainable behavior.

## Methods

### Study Design

A cross-sectional, quantitative research design was adopted to investigate the relationships among decent work practices, professionalism, and sustainable behavior in line with the “Strengthening the Reporting of Observational Studies in Epidemiology” guidelines.

### Participants and Setting

We recruited a convenience sample of clinical staff nurses from Mansoura University Hospital in Mansoura, Egypt. The inclusion criteria were: (1) willingness to participate, (2) holding a valid Egyptian nursing license, and (3) active involvement in direct patient care for at least the past year. Nursing students, interns, and nurses in managerial positions were excluded from the study.

### Sample Size

G*Power 3.1.9.7 (Faul et al., 2007) was used to calculate the sample size based on a linear multiple regression model with a fixed model and a single regression coefficient. The calculation assumed a two-tailed test, anticipating a medium effect size of 0.1, with an alpha level (α) of .01 and a power of 0.95. As the model includes 10 predictors, that is, 8 sociodemographic variables, one mediator, and one dependent variable, a minimum sample size of 182 participants was required. After factoring in a potential dropout rate of 30%, a study sample of 237 nurses was targeted. Of the 237 nurses initially invited to participate, 206 returned valid questionnaires, representing the final sample (effective response rate: 86.9%).

### Data Collection Tools

#### Introductory information form

This form was used to collect information on participant age, gender, marital status, residence, educational degree, working department, years of professional experience, and work shift.

#### Decent Work Scale (DWS)

The Arabic version of the DWS, originally developed by Duffy et al. (2017), was utilized to assess the participants’ level of decent working conditions. The Arabic version, previously translated and validated across three studies, has demonstrated robust psychometric properties in the Arabic cultural context (El-Gazar, Shawer, et al., 2024; El-Gazar & Zoromba, 2024; Zoromba et al., 2024). The DWS comprises 15 items organized into five dimensions of three items each: (I) physically and interpersonally safe working conditions, (II) access to health care, (III) adequate compensation, (IV) hours that allow for free time and rest, and (V) organizational values that complement family and social values. Responses are measured on a 7-point Likert scale, with 1 denoting “*strongly disagree*” and 7 denoting “*strongly agree*”. The scale includes four reverse-coded items to ensure response consistency. Higher total scale scores indicate a greater degree of satisfaction with decent work conditions. The original version of this scale yielded good internal consistency, with a Cronbach’s α of .86. In this study, the Cronbach’s α of .91 is consistent with excellent reliability.

#### Nurses’ Professionalism Inventory (NPI)

The Arabic version of the NPI, originally developed by Ichikawa et al. (2020), was employed to assess the level of professionalism in nurses. This version was translated, adapted, and validated for use in this study. The NPI comprises 28 items distributed across the five subscales of (I) accountability, (II) self-improvement, (III) professional attitude, (IV) advancement of the nursing profession, and (V) professional membership. Each item is rated on a 6-point Likert scale ranging from 1 (*strongly disagree*) to 6 (*strongly agree*), with higher scores indicating stronger professionalism. In its original form, the NPI has demonstrated excellent psychometric properties, and construct validity is supported through factor analysis, confirming the intended structure of its five subscales and indicating an internal consistency reliability ranging from .84 to .90 across the subscales.

Face, content, and construct validity of the Arabic version of the NPI were assessed in consultations conducted with a panel of seven experts consisting of four nurse academicians and three clinical nurses. The expert panel evaluated the extent to which the items accurately capture the intended constructs to establish face validity. A 4-point Likert scale, with 1 denoting “*not relevant*” and 4 denoting “*highly relevant*, ” was used to assess content validity, with a scale-level CVI/average (S-CVI/Ave) ≥.90 and item-level content validity index (I-CVI) ≥.78 indicating content validity to be satisfactory. The experts confirmed the face and content validity of the Arabic version of the NPI, with I-CVI values ranging from .92 to 1.00 and the S-CVI/Ave recorded at .94, indicating excellent content validity. In this study, the Arabic version of the NPI demonstrated excellent internal consistency reliability, with a Cronbach’s α of .93.

#### Self-Reported Sustainable Behavior (SRSB) Scale

The Arabic version of the SRSB scale, originally developed by Avelar and Farina (2022), was employed to measure sustainable behavior across the environmental, social, and economic dimensions. This 15-item scale was adapted and validated for use in assessing sustainable behavior related to environmental awareness, social responsibility, and economic mindfulness in nurses. Each item is scored on a 7-point Likert scale ranging from 1 (*strongly disagree*) to 7 (*strongly agree*), with higher scores indicating stronger sustainable behavior engagement. The original English version of the SRSB demonstrated good psychometric properties, while construct validity was further confirmed through factor analysis, supporting the three-dimensional structure of the scale. A Cronbach’s α of .86 demonstrates good reliability.

For this study, the Arabic version of the SRSB underwent face, content, and construct validity assessments. A panel of seven experts, including four academic researchers and three clinical nurses, was invited to review the scale to ensure it accurately captures the targeted constructs. Face validity was confirmed using expert feedback, and content validity was assessed using a 4-point Likert scale (1=*not relevant* and 4=*highly relevant*), with items deemed valid if their S-CVI/Ave value was ≥ .90 and I-CVI value was ≥ .78. The expert panel confirmed scale validity, with I-CVI scores ranging from .94 to 1.00 and an S-CVI/Ave of .96. Thus, the assessed reliability of this scale was identified as excellent, with a Cronbach’s α of .93.

### Pilot Study

A preliminary test was conducted on a sample of 20 nurses recruited from the hospital targeted in this study. The main goals of this test were to evaluate the feasibility and clarity of the survey questions. The feedback from respondents identified the survey items as clear and easily comprehensible.

### Data Collection

Data were collected using an online survey hosted on Google Forms, conducted between October and December 2024. Participants accessed the survey by scanning a QR code shared through departmental and hospital social media platforms. These QR codes were disseminated with the approval of hospital administrators and unit managers. Upon accessing the survey, nurses were presented with an information sheet that explained the voluntary and anonymous nature of this study, followed by a request for their consent to participate. To ensure sufficient participation, reminders including a hyperlink to the survey and additional study details were sent via email and private messages on platforms such as Facebook Messenger and WhatsApp 2 weeks after the initial contact. To maintain response integrity, all of the survey questions were designated as mandatory, and the survey system was configured to allow only one submission per IP address. Data collection concluded when the number of valid replies exceeded the calculated minimum sample size.

### Statistical Analysis

Data were analyzed using IBM SPSS Statistics version 27 (IBM Corp., Armonk, NY, USA) supplemented by the PROCESS macro developed by Andrew F. Hayes (version 4.2). Descriptive statistics, including mean, standard deviation, and frequency, were calculated to summarize the characteristics of the sample. Normality was addressed, with the results indicating the data were approximately normally distributed. To examine the relationships between the variables, Pearson correlation coefficients were computed. Also, independent samples *t*-tests and one-way ANOVA were conducted to explore differences in mean scores of the studied variables across various sociodemographic characteristics. For mediation analysis, the PROCESS macro (Model 4) was applied to test the hypothesized mediation model in which professionalism was examined as a mediator in the association between decent work practices and self-reported sustainable behavior. The mediation model was tested using bootstrapping procedures with 5,000 bootstrap samples. Significance was determined at the 95% confidence level, and *p-*values <.05 were considered statistically significant.

### Ethical Considerations

This study was approved by the Standing Committee of Bioethics Research, Prince Sattam bin Abdulaziz University (No. 351/2024) and was implemented in line with the principles of the Helsinki Declaration. All of the participants were fully informed of the study objectives and provided online informed consent before participation. Nurses were informed that their contribution was completely voluntary and were reminded not to include any personal data on the assessment to maintain anonymity.

## Results

As presented in Table 1, participants under 30 years old and those over 30 years old scored similarly across all three variables. Male participants had slightly higher mean scores for decent work practices, professionalism, and sustainable behavior than their female counterparts. Participants living in rural areas scored marginally higher on all three variables than their urban peers. Participants who were single scored slightly higher across all three variables than their married counterparts. Those with a Bachelor of Science degree reported higher scores in decent work practices, professionalism, and sustainable behavior than those with a technical education. Those working in inpatient departments had slightly higher scores than those working in ICU/ER and outpatient departments. Those with less than 5 years of experience reported marginally higher scores in sustainable behavior than those with more than 5 years of experience. Finally, those working night shifts reported slightly higher scores for all three variables than those working daytime only or day and night shifts. In summary, while sociodemographic characteristics were found to influence scores, none reached statistical significance.

**Table 1:** Relationship Between Sociodemographic Characteristics and Mean Variable Scores (*N*=206)

Characteristic		Decent Work Practices		Professionalism		Self-Reported Sustainable Behavior	
	*n* (%)	*M (SD)*	*t/F*	*M (SD)*	*t/F*	*M (SD)*	*t/F*
Age (years) ^a^			0.613		0.511		0.737
<30	119 (57.8)	5.34 (0.77)		4.74 (0.64)		4.04 (0.45)	
≥30	86 (42.2)	5.40 (0.63)		4.78 (0.60)		3.99 (0.47)	
Sex ^a^			0.597		0.847		1.529
Male	45 (21.8)	5.41 (0.47)		4.81 (0.39)		4.09 (0.28)	
Female	160 (78.2)	5.35 (0.77)		4.74 (0.67)		4.00 (0.50)	
Residence			0.704		0.642		0.314
Urban	62 (30.1)	5.29 (0.87)		4.71 (0.71)		4.00 (0.54)	
Rural	144 (69.9)	5.37 (0.69)		4.78 (0.58)		4.03 (0.42)	
Marital ^a^			1.133		1.507		1.733
Single	41 (19.9)	5.46 (0.52)		4.85 (0.37)		4.10 (0.30)	
Married	164 (80.1)	5.34 (0.76)		4.74 (0.67)		4.00 (0.49)	
Degree			1.078		2.375		1.447
Technical	181 (87.9)	5.35 (0.75)		4.74 (0.65)		4.01 (0.48)	
BSc	25 (12.1)	5.45 (0.36)		4.90 (0.22)		4.10 (0.23)	
Department			*F*=0.355		*F*=0.524		*F*=0.960
Inpatient	73 (35.4)	5.43 (0.72)		4.82 (0.60)		4.02 (0.47)	
ICU/ER	110 (53.4)	5.33 (0.73)		4.72 (0.60)		4.03 (0.44)	
Outpatient	23 (11.2)	5.19 (0.89)		4.71 (0.75)		3.99 (0.53)	
Experience (years)			0.043		0.120		1.178
<5 years	86 (41.7)	5.35 (0.72)		4.75 (0.58)		4.06 (0.38)	
≥5 years	120 (58.3)	5.35 (0.78)		4.76 (0.65)		3.99 (0.51)	
Working shift			*F*=0.594		*F*=0.215		*F*=0.488
Day	75 (36.4)	5.31 (0.96)		4.70 (0.82)		3.99 (0.62)	
Night	101 (49.0)	5.47 (0.36)		4.93 (0.22)		4.11 (0.22)	
Both	30 (14.6)	5.34 (0.66)		4.75 (0.51)		4.01 (0.36)	

*Note.* BSc = Bachelor of Science; ICU = intensive care unit; ER = emergency room.

^a^ Missing data.

As shown in Table 2, the correlation analysis revealed significant positive relationships between all of the studied variables at the 0.01 level. Specifically, decent work practices were shown to correlate strongly with professionalism (*r*=.870) and self-reported sustainable behavior (*r*=.897). Similarly, professionalism correlated significantly with self-reported sustainable behavior (*r*=.823). These correlations suggest that higher levels of decent work practices are associated with higher levels of professionalism and sustainable behavior among nurses.

**Table 2:** Means, Standard Deviations, Reliability, and Correlations for the Studied Variables (*N*=206)

Variable	Mean (*SD*)	α	1	2	3
Decent work practices	5.36 (0.72)	91	1		
Professionalism	4.76 (0.62)	.93	.870^**^	1	
Self-Reported Sustainable Behavior	4.02 (0.46)	.84	.897^**^	.823^**^	1

^**^ Correlation is significant at the .01 level (two-tailed).

**Figure 1:**
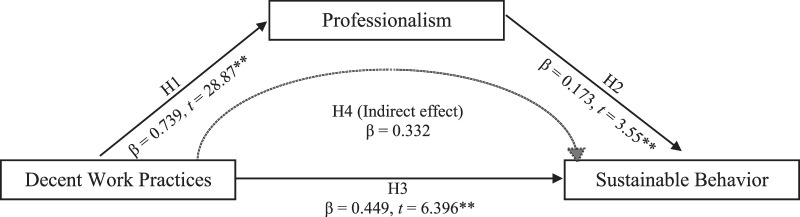
Estimates of the Mediation Model (*N*=206)

As shown in Table 3 and Figure 1, a significant and positive association was found between decent work practices and professionalism (β=0.739, *p*<.001), indicating that when nurses perceive their work environment as decent, their level of professionalism increases. In addition, decent work practices directly and positively impacted self-reported sustainable behavior (β=0.173, *p*<.001), suggesting a better work environment encourages nurses to engage in sustainable practices in their daily work activities. The other direct relationship between professionalism and their self-reported sustainable behavior was statistically significant (β=0.449, *p*<.001), suggesting higher levels of professionalism are associated with increased likelihood of engaging in sustainable practices in their daily work activities. Furthermore, the analysis identified a significant indirect effect, through professionalism, of decent work practices on sustainable behavior (β=0.332). This finding indicates professionalism plays a critical mediating role in the translation of decent work practices into sustainable behavior in nurses.

**Table 3:** Mediation Model Estimates (*N*=206)

Effect	β	*SE*	*t*	*p*	95% CI
Direct effect					
Decent work practices → professionalism	0.739	0.026	28.87	<.001	[0.689, 0.789]
Decent work practices → Self-Reported Sustainable Behavior	0.173	0.049	3.55	<.001	[0.077, 0.269]
Professionalism → Self-Reported Sustainable Behavior	0.449	0.059	6.40	<.001	[0.332, 0.566]
Mediated indirect effects					
Decent work practices → Self-Reported Sustainable Behavior via professionalism	0.332	0.063			[0.194, 0.447]

*Note.* β = coefficient; *SE* = standard error; CI = confidence interval.

## Discussion

This study was designed to investigate the relationships among decent work practices, professionalism, and sustainable behavior in the context of nurses, with a specific focus on the mediating role of professionalism.

### Decent Work and Professionalism

The positive relationship observed between decent work practices and professionalism aligns with previous research, emphasizing the importance of supportive work environments in fostering professional behavior among nurses. Al Sabei et al. (2021) demonstrated that nurses who recognize their work environment as supportive are more likely to exhibit high levels of professionalism. The findings of this study extend this understanding by highlighting the specific role of decent work practices, for example, fair compensation, safe working conditions, and chances for professional development, in enhancing self-perceived professional identity in nurses.

Decent work environments provide the resources and support necessary to enable nurses to engage in ethical practice, continuous learning, and advancing the nursing profession. These environments contribute to job satisfaction, which is a critical determinant of professional engagement (El-Gazar, Abdelhafez et al., 2022; Mabona et al., 2022; Zoromba et al., 2024). When nurses feel valued and supported in their roles, they are more likely to adhere to the standards of their profession and take pride in their work, both of which are key aspects of professionalism (Cao et al., 2023; El-Gazar et al., 2023). In the context of clinical practice, decent work conditions are an essential resource in fostering professional identity. This professional identity, in turn, motivates nurses to strive for excellence in their practice and contribute to the overall quality of care provided to patients.

### Professionalism and Sustainable Behavior

The significantly positive relationship found between professionalism and sustainable behavior in this study echo Luque-Alcaraz et al. (2024), which found nurses who prioritize professionalism to be more likely to adopt sustainable behaviors such as reducing energy use and minimizing waste in clinical settings. These behaviors are integral to environmental sustainability in health care. Nurses who demonstrate high levels of professionalism are concerned not only with providing high-quality care but also with the long-term well-being of patients, communities, and the environment (Cao et al., 2023; Oldland et al., 2020). This commitment to practice drives nurses to engage in sustainable behaviors, as they recognize the importance of conserving resources and reducing the environmental impact of health care practices. Moreover, professionalism is associated with a continuous commitment to learning and improvement, encouraging nurses to stay informed about best practices and integrate these practices into their daily work (Eraut & Hirsch, 2007). This commitment to professional development aligns with the principles of sustainability, as it promotes a culture of continuous improvement and long-term thinking in health care.

### Impact of Decent Work on Sustainable Behavior

The direct and positive impact of decent work on sustainable behavior observed in this study is consistent with previous research showing that the provision of decent work conditions, such as safe working environments, adequate compensation, and work-life balance is essential to promoting sustainable behavior in health care (Ariza-Montes et al., 2019). Decent work environments provide nurses with the resources necessary to engage in sustainable practices. These practices are not only beneficial to the environment but also to the long-term viability of health care resources and the well-being of patients and communities.

The findings of this study suggest decent work practices that promote equity and social justice also encourage nurses to advocate for practices that promote health equity and social sustainability in their communities. This aligns with Bahlman-van Ooijen et al. (2023), who found that nurses who experience decent work conditions are more likely to be motivated to engage in behaviors that are not only beneficial for their immediate work environment but also for the broader health care system and society.

The novel approach taken in this study to assessing the mediating role of professionalism in the relation between decent work and sustainable behavior provides important insights into the mechanisms through which decent work influences sustainable behavior in nursing.

The mediating role of professionalism is supported by job resources theory, which posits that resource availability in the workplace enhances motivation and engagement in employees, leading to positive work outcomes (Demerouti & Bakker, 2023). In the context of nursing, decent work conditions represent a resource for enhancing professionalism, which in turn leads to sustainable behavior. Furthermore, the theory of planned behavior holds that subjective norms, attitudes, and perceived behavioral control affect individual intentions and behaviors (Ajzen, 1991). In the context of nursing, professionalism may be seen as a positive attitude that influences intentions to practice sustainable behaviors. Decent work conditions provide the support and resources necessary to shape these positive attitudes, thus mediating the relationship between decent work and sustainable behavior.

### Study Implications

#### Theoretical contributions

This study offers important theoretical contributions to the existing literature on nursing, decent work, professionalism, and sustainability. First, in integrating these constructs into a comprehensive model, a deeper understanding of the multifaceted relations among work conditions, professional identity, and sustainability in health care is provided. Second, this study extends the application of job resources theory and the theory of planned behavior to the nursing profession, demonstrating how these theories may be used to explain the relationships among decent work, professionalism, and sustainable behavior.

Furthermore, the findings contribute to the growing body of literature linking professionalism to sustainability in the context of health care. By demonstrating professionalism as a significant predictor of sustainable behavior in nurses, this study suggests that efforts to promote professionalism in nursing can positively impact sustainability in health care practices. This finding has important implications for the future development of interventions that promote sustainability in health care.

#### Practical implications for health care management

First, the findings highlight the importance of creating work environments that support decent work practices. In providing safe working circumstances, fair compensation, and chances for professional development, health care organizations can enhance professionalism in their nursing staff and encourage their engagement in sustainable practices, ultimately contributing to the long-term sustainability of health care resources as well as patient and community well-being.

Second, in fostering professionalism, health care organizations can maximize the impact of decent work practices on the sustainability of health care practices. Third, this study provides valuable insights for health care managers and policymakers seeking to promote sustainability in health care. By understanding the complex relationships among decent work, professionalism, and sustainable behavior, health care organizations can develop targeted interventions that promote health care sustainability.

Building on the findings in this study, future research should explore the longitudinal relationships among decent work, professionalism, and sustainable behavior. Furthermore, research into specific interventions designed to enhance professionalism and sustainable behavior in nursing can provide practical recommendations to health care managers and policymakers.

### Limitations

While this study makes important contributions to the literature, several limitations should be considered. First, the cross-sectional design used limits the potential to draw causal inferences regarding the relationships among decent work, professionalism, and sustainable behavior. Second, this study depended on self-reported data, which may be influenced by social desirability bias. Thus, the participants may have reported higher levels of professionalism and sustainable behavior than they actually exhibit in practice. Third, this study was conducted in a specific geographical region within a specific type of health care institution, limiting the generalizability of the findings to other regions and health care settings. Finally, although the quantitative approach used to examine the relationships among decent work, professionalism, and sustainable behavior provided valuable insights into these connections, qualitative and mixed-method designs may offer more in-depth understandings.

### Conclusions

The findings of this study provide valuable insights into the complex relationships among decent work, professionalism, and sustainable behavior in nursing. By demonstrating the significant impact of decent work practices on professionalism and sustainable behavior, this study highlights the importance of creating supportive work environments that promote professionalism and sustainability in health care. The findings have important implications for health care management and policy, suggesting that efforts to promote decent work and professionalism can enhance the sustainability of health care practices. As the global trend toward sustainability continues, understanding the factors that enable nurses to contribute effectively to this goal is essential for the long-term viability of health care systems worldwide.
